# Traumatic brain injury and sight loss in military and veteran populations– a review

**DOI:** 10.1186/s40779-021-00334-3

**Published:** 2021-07-28

**Authors:** Syeda F. Hussain, Zara Raza, Andrew T. G. Cash, Thomas Zampieri, Robert A. Mazzoli, Randy H. Kardon, Renata S. M. Gomes

**Affiliations:** 1 Research & Innovation, Blind Veterans UK, 12-14 Harcourt Street, London, W1H 4HD UK; 2 Bravo Victor, Research, 12-14 Harcourt Street, London, W1H 4HD UK; 3 Blinded Veterans Association, 1101 King Street, Suite 300, Alexandria, Virginia 22314 USA; 4 Department of Ophthalmology, Madigan Army Medical Center, 9040 Jackson Avenue, Tacoma, Washington, 98431 USA; 5 Iowa City VA Health Care System and Iowa City VA Center for the Prevention and Treatment of Visual Loss, Iowa City, Iowa 52246 USA; 6 Department of Ophthalmology and Visual Sciences, The University of Iowa, Iowa City, Iowa 52242 USA; 7 Northern Hub for Veterans and Military Families Research, Department of Nursing, Midwifery and Health, Faculty of Health and Life Sciences, Northumbria University, Newcastle, NE7 7XA UK

**Keywords:** Traumatic brain injury, Visual impairment, Military, Veteran

## Abstract

War and combat exposure pose great risks to the vision system. More recently, vision related deficiencies and impairments have become common with the increased use of powerful explosive devices and the subsequent rise in incidence of traumatic brain injury (TBI). Studies have looked at the effects of injury severity, aetiology of injury and the stage at which visual problems become apparent. There was little discrepancy found between the frequencies or types of visual dysfunctions across blast and non-blast related groups, however complete sight loss appeared to occur only in those who had a blast-related injury. Generally, the more severe the injury, the greater the likelihood of specific visual disturbances occurring, and a study found total sight loss to only occur in cases with greater severity. Diagnosis of mild TBI (mTBI) is challenging. Being able to identify a potential TBI via visual symptoms may offer a new avenue for diagnosis.

## Background

With the development of powerful explosives that have significant fragmentation ability, the incidences of eye injuries acquired by military personnel rose greatly from the 19th to the twentieth century. Up until World War II, incidences of war-related eye injuries were less than 2% and rose to 13% in Operation Desert Storm (1991) [1]. Whilst the rates of eye injuries have increased over time, especially as the methods and tactics of using explosives on the battlefield have changed, the occurrences of ocular injuries, vision dysfunction and vision impairment remain a long-time staple of warfare. In the last half century, the use of explosive devices has become more common with the rise in regional and global terrorism, thus significantly affecting people in civilian settings as well. Ocular injury incidence rates in the Manchester terror attacks in 2017, for example, were 3% [1]. As a consequence of the widespread use of explosives, not only has the number of military service members with ocular injuries increased but also the number of cases of blast-induced traumatic brain injury (TBI) has increased. Visual dysfunction and sight loss do not just occur as a result of direct physical damage by the heat, debris and fragments released by explosives, but are also common after TBI [2]. This review primarily focuses on visual symptoms as a result of TBI.

## Search strategy, selection and inclusion criteria

Searches of PubMed, Scopus and Google Scholar were performed to obtain the data and articles for this review. Abstracts and articles were reviewed and included if they met criteria for discussing sight loss and/or visual dysfunctions amongst the military or veteran population as a result of TBI (any cause). There was no date limitation to the articles included. As this is primarily a narrative review, there were no strict inclusion or exclusion criteria, however the criteria for including experimental studies were: subjects of the study or sample group within the study had a diagnosis of a TBI or comparable head injury (blast or non-blast cause); visual complaints occurring only after a TBI/head injury had occurred, published in the English language and in a peer reviewed journal. Studies were not excluded if data analysis was conducted using retrospective patient data (this is the case with most studies that look at the effects of historical TBI), or if TBI severity was not classified (in some patient records severity is not always recorded). Any form of visual dysfunctions or complaints were considered - whether they were assessed via screening methods or self-reported. There was no restriction regarding subject age, gender, location of study participants, type of military service, or location or duration of military service.

There is a vast amount of literature that uses different terms when referring to TBI for example ‘shell shock’ or ‘acquired brain injury’, or ‘closed head injury’, and so on. Our search criteria used the more current and well-known terminology ‘traumatic brain injury’. By doing so, however, we may have focused more on recent conflicts which occurred after the two world wars. In addition, there is now greater understanding of TBI than when the phenomenon was first discovered.

## History of TBI

The phenomenon of ‘traumatic brain injury’ has been known about from as early as World War I (1914–1918) although it was referred to by a different name. Soldiers at the time had been experiencing unusual symptoms as a result of exposure to artillery barrage. Many experienced symptoms such as amnesia, headache, inability to concentrate, tinnitus, and hypersensitivity to noise [3]. The term ‘shell shock’ was coined to describe these cases. There was no real definition for ‘shell shock’ nor was there an agreed understanding of pathology [4]. It became further complicated as many who were close to the explosion site did suffer from some of these symptoms, but did not have any obvious head wounds [5]. Puzzlingly, soldiers also had other symptoms such as anxiety, sleep disturbance, depression, and even suicidal behaviour [6]. It was difficult to distinguish between the psychological aspects that were being presented, but the difference became apparent when comparing physical symptoms. It is now apparent that the set of symptoms that were being referred to as ‘shell shock’ are symptoms that are commonly found in those with TBI and post-traumatic stress disorder (PTSD).

Injuries as a result of exposure to blasts can be categorised as primary, secondary, tertiary, quaternary or quinary. Primary injuries result from the over-pressurisation wave created by the explosion - sometimes referred to as primary blast injuries. A secondary blast injury occurs due to flying debris or shrapnel put in motion by the forceful winds created by a blast wave, e.g. metal casing or objects from the explosive device and local material disturbed by the blast. The flying objects can lead to either penetrating or blunt TBI. Tertiary blast injuries occur as a result of a displaced body impacting with a stationary, solid object [6]. Quaternary injuries occur due to exposure to heat and light from the explosion as well as inhalation of toxins and gases resulting in injuries such as thermal burns and respiratory problems. Quinary injuries occur as a result of exposure to toxic materials following an explosion, for example, bacteria and radiation [7]. Blast injuries in the military setting can occur due to a wide variety of weapons, including improvised explosive devices (IED), mines, mortars and rocket-propelled grenades.

Whilst the use of explosives, and therefore also blast-related TBI, are commonly associated with the military, TBI is not solely caused by explosives in this setting. TBI, particularly mild TBI may occur from falls, gunshots, physical training exercises, athletics, recreational sports, and motor-vehicle accidents [8].

## Epidemiology of TBI

Between 2000 and 2020, during which both Operation Iraqi Freedom (OIF) and Operation Enduring Freedom (OEF) took place (2003–2011, and 2001–2014 respectively), the total number of service members worldwide who were recorded as having had TBI was 430,720 [9]. As previously mentioned, the number of service members being diagnosed with brain injuries has risen due to the increased use of explosives such as IED, but also because there is a greater likelihood of service members surviving events that can cause brain injuries. There is better access to exceptional medical care and better protective equipment, improving the chances of survival [10].

Ultimately, however, it is difficult to get a true appreciation of the numbers of military service members that acquire a TBI due to inability to promptly diagnose an injury in a combat environment. After regaining consciousness, the injured soldier may assume that they are fine and may not even realise, let alone report, that they have suffered a head injury. There may also be an inclination to ignore any symptoms and simply carry on with duty. To address this, there is the need to implement event-based screening [11]. Underreporting may also be as a result of differences in TBI definitions, classifications and incorrectly ascribed medical codes. Relying on self-reported data from surveys can also be problematic as it is subject to bias. The individual may not be able to recall details of the incident or may even withhold certain information in order to accelerate return to duty [12]. Some of these factors may also affect reporting of TBI amongst the civilian population. The deployment period can differ in length depending on the country and service from which the personnel are deployed; TBI rates reflect deployment length, with the U.S. (which routinely has longer deployments) having the highest global average [13], the UK [14] and Canada having lower numbers. In addition, every country reports TBI epidemiology according to its own set of surveys, tools and diagnostic definitions [11].

## TBI to vision loss: pathophysiological pathways and biomechanical mechanisms

The principal stages of a TBI are the primary insult and secondary insult. The primary insult is the mechanical portion of the injury which results in acute symptoms such as tissue loss, haemorrhaging and hematoma. The secondary insult is the physiological response that is triggered by the primary insult and can occur over a delayed period - minutes, hours or days after the initial injury. Secondary lesions include inflammation, blood-brain barrier disruption and oedema [15]. The primary insult may result in a focal or diffuse injury (both can occur at the same time in moderate-severe cases), but a common feature of TBI is diffuse axonal injury (DAI) which occurs in 70% of TBI cases [16]. Whether the injury is focal or diffuse depends on the actual mechanism of injury, e.g. if it is penetrating or a closed head trauma it will more likely be focal, but if the injury is non-contact, i.e. from the overpressure of a blast wave, then it will be more diffuse. For the case of diffuse injury, there is shearing and stretching of neuronal axons, oligodendrocytes and blood vessels which can cause cerebral oedema and ischemia. There is axonal damage in the subcortical and deep white matter tissue, and the extent to which the axons are damaged can determine the severity of the brain injury. Traumatic axonal injury is what leads to cognitive impairment and decline as white matter pathways are disrupted [17]. With the secondary insult, there are several key pathophysiological events taking place which result in secondary injuries. These include: neuroinflammation, axonal degeneration, apoptosis of neurons and oligodendrocytes to name a few [16]. Targeting some of these mechanisms with therapeutic treatments may be a manner of preventing secondary injuries from accelerating cognitive decline following a TBI. Interestingly, a study using Wallerian degeneration slow strain (*WldS*) mice, showed resistance to axonal degeneration following a blast TBI. The mutation in this strain was even found to protect the mice from visual dysfunction [18].

To gain insight into how a TBI may gradually, or in some cases almost instantly [19], lead to visual dysfunctions or vision impairment it may be helpful to understand what is occurring at a cellular and molecular level. Determining the pathophysiological mechanisms which occur following a TBI event and modelling the primary and secondary insults may be useful in developing treatments. However, TBI pathology is complex and depends on factors such as severity and how focal or distributed the injury is. Animal models, usually rodents placed in shock tubes or exposed to direct air-blasts aimed at either the head or the globe, offer an avenue to simulate isolated aspects and impacts of blasts, e.g. the effect of heat and radiation, torsional and rotational forces, focal blast impact, the pressure from the primary blast wave. In fact, much of what we already know about the impact of blasts, the progression of TBI and the secondary visual effects are from rodent models. There are obvious limitations with using rodents, for example, differences in brain size and structure (lack of gyri and sulci in the rodent brain) [20], the injury may progress at a different rate or timetable [20], limited genetic variation in rodent models, and the typical difficulties in animal to human translation. However, the key aspect is that, on a cellular level, there are similarities between a rodent and a human brain [21]. Computational models can offer an alternative simulation method for the effects of blasts on the brain and ocular system. One such example is the use of the finite element method (FEM) to demonstrate how a blast wave propagates through the orbital cavity and affects the globe [22–24]. One FEM study showed how each eye structure was affected by the pressure created in the orbit by blast. The angle at which the blast wave propagates towards the orbit and the orientation of the orbit determine the pressure at certain points in the eye. The use of 2.5 mg of Trinitrotoluene (TNT) at 0.5 m distance is able to produce a high enough pressure to damage the choroid, retina and optic nerve. The cornea and vitreous base reach peak pressure and so are easily subject to damage. The geometry of the orbit and certain structure densities influence the pressure created by the inbound wave from the explosive and the reflected waves within the orbital cavity. The type of interface, i.e. bony versus fluid interface, influences how the wave reflects [22]. Studies have also used axisymmetric 3-dimensional models to mimic the eye [25], and this means that as advancements are being made in modelling, understanding of the pathophysiology and mechanobiology of vision impairment is set to improve. Models are heading in the direction of stem cells, humanised models, organoids and organ-on-chip models [26, 27]. Organoids attempt to mimic organs; they are made of stem cells which are organ-specific, and are able to self-organise and self-assemble to form an organ’s 3-dimensional architecture [28] such as eye structures (lens, cornea, retina) [27]. Organ-on-chip models imitate organs at a micro-level. These are microchips which are transparent and contain hollow microfluidic channels and cell compartments, lined with living human organ-specific cells. As they are transparent, when external artificial forces are applied to replicate the organ’s physical environment, the effect on the cells can be seen [27]. These technological advancements mean that the issue of clinical translatability which arises from using in-vitro or in-vivo methods may be bypassed [27], and more readily accessible high-resolution advanced imaging may be available [29] to observe and investigate the mechanisms that cells undergo during vision impairment. Furthermore, they may offer opportunities to further develop targeted treatments. These models may in the future provide a more viable study method for how certain eye structures undergo changes following a TBI, in comparison to existing models which have their own limitations.

### Mechanism of injury to optic nerve

The use of polycarbonate eyewear as part of military protective equipment has been able to protect against certain types of injuries, for example, ballistic ocular trauma [30], however traumatic optic neuropathy (TON) remains highly prevalent following a TBI with a reported incidence rate of 0.5–5.0% in cases of closed head injuries [31–33]. This injury to the optic nerve can lead to vision impairment alongside loss of consciousness. A longitudinal study found that even when controlling for comorbidities, during a ten-year follow up period the incidence risk of optic neuropathy was over 3 times greater in those with TBI in comparison to controls (*HR* = 3.017, 95% CI 2.767–3.289, *P* < 0.001) indicating that TBI is a risk factor for optic neuropathy. Rodent models have been used to demonstrate the pathophysiology of mTBI and optic neuropathy. Histopathological and immunohistochemistry tests have shown that damage to the optic nerve often occurs after a mTBI, leading to vision complications [34, 35]. It was suggested that in order to prevent vision loss (where possible), regular eye examinations following a TBI event are important [36]. Urosevich et al. [37], also recommend extra caution with veterans, in that they should be checked for any underlying issues with the structure and function of the visual system. Additionally, before carrying out eye checks, ophthalmologists should have access to patient history and information regarding periods of deployment and whether there were any previous head injuries or exposure to blasts.

There can be direct or indirect TON. A direct TON is when the optic nerve is directly damaged so it can no longer function effectively. It may be anatomically damaged such that there is avulsion or transection. It may also occur because the optic canal is becoming fractured [38]. A direct injury may occur as a result of penetration by airborne fragments ejected from the explosion site. Indirect TON is the functional or anatomic damage to the optic nerve through the transmission of energy or forces from a region distant to the nerve, e.g. the supraorbital ridge or the fronto-temporal region. However, there is no damage to the ocular and cerebral tissue [33, 39]. Indirect TON can occur when there is concussive trauma to the forehead [40, 41] or a blast-TBI. The neuropathology of an indirect TON can be distinguished from a direct TON [42]. Indirect injuries are most likely to result in vision loss when the intracanalicular portion, the most vulnerable region of the optic nerve, is damaged as a consequence of the transmission of shearing forces to the blood supply to the optic nerve and increases in intracanalicular pressure [32, 43]. Bernardo-Colón et al. [42] showed through blast modelling, that there is short term elevation of intraocular pressure (IOP), loss of retinal ganglion cells (RGC) and axonal degeneration along the length of the optic nerve. FEM modelling showed damage to the optic nerve from blast pressure exposure [22], and central fluid percussion injury (cFPI) modelling using mice showed evidence of axonal injury in the optic nerve [44].

A 3-dimensional model of the human eye demonstrated how the optic nerve may undergo degeneration if it experiences strain beyond its range. This TBI simulation showed that the optic nerve experiences a high strain rate, suggesting that it is vulnerable to damage [25]. This study in particular highlights that cerebrospinal fluid (CSF) pressure needs to be taken into account when considering the biomechanical aspect of the optic nerve which other models do not often do. The simulation showed a transient increase in both IOP and CSF pressure [25]. In Weichel’s study [45], one of the most common causes of vision impairment was TON. Of the 523 eyes studied in Weichel’s investigation, 20% experienced TON; 11% had a direct injury, of which some optic nerves were completely avulsed or transected (2% of the 523 eyes). There were also indirect injuries, although fewer than the number of direct injuries (9%). In general however, indirect TON is more commonly occurring than direct TON [41].

Diagnosing a TON is challenging. It is easier to diagnose a direct TON as there is usually - but not always - an open wound, and imaging methods such as computed tomography and magnetic resonance imaging can be used [41]. Imaging techniques such as optical coherence tomography (OCT) will show thinning of the retinal ganglion cell layer within weeks and retinal nerve fibre layer (RNFL) within months due to retrograde degeneration. Ophthalmologic functional tests (e.g. visual acuity, relative afferent pupil defect, and visual field tests) may be useful in diagnosing TON [46]. In some cases, MRI and CT imaging of the orbital and canalicular portion of the optic nerve may show evidence of acute damage. With indirect TON, some degree of visual recovery is possible with an improvement rate of 40–60%. In contrast, the possibility of regaining vision following a direct TON is not so promising as vision loss is likely to be irreversible [47].

### Mechanism of injury to globe and retina

RGC are peripheral neurons connected to the central nervous system. RGC are found near the inner surface of the retina and the axons from these cells extend to form the optic nerve. RGC damage and dysfunction can lead to chronic visual impairment, thus targeting RGC in order to preserve them and maintain normal functioning could provide a possible preventive measure for long term TBI-related vision loss.

Evans et al. [48] showed that different types of TBI, i.e. blast versus blunt, can elicit different phenotypic changes within the eye which are capable of leading to visual dysfunction or loss. In the blast model, there were signs of reduced RGC, posterior vitreous detachment, vitreous haemorrhage, photoreceptor degeneration and subretinal haemorrhage. The blunt model (lateral fluid percussion injury) resulted in anterior uveitis but no difference in the number of RGC as seen in the blast case. Tzekov et al. [34] saw damage to both optic nerve and RGC as well as thinning of the inner retina following repetitive blunt TBI. Molecular changes such as these, which dependently change according to injury type, may be useful as biomarkers.

Pattern electroretinography (PERG), a technique used to measure RGC and optic nerve damage, and immunohistochemistry tests indicate death and depletion of RGC [49, 50]. Dutca et al. [50] found that RGC injury and dysfunction begin at approximately 4 weeks after the initial blast injury. The injury demonstrated in this model is diffuse where the blast is delivered to the head and not the eye directly. It is important to distinguish between blast models which may use different modes of blast injury that may target different head parts.

Along with degradation of RGC, vision may also be lost due to retinal scarring. Scarring occurs as a result of retinal haemorrhaging when the blood vessels in the eyes transmit blood at a much higher than normal pressure in response to being exposed to the high impact forces from a blast [46, 51].

Explosives may lead to irreversible vision loss through direct damage to the globe as well. Open globe injuries may be classified as laceration (perforation, penetration, and intraocular foreign body) or rupture. Preserving vision depends on several factors including the severity of the initial trauma, location of the trauma (which ocular structures are involved), injury mechanism, whether there is infection, and being able to diagnose and treat trauma promptly [52]. Further to secondary damage by fragments ejected from an explosion, primary blast waves themselves can damage the visual organ as found by DeMar et al. [53]. In this study, rats were used to model the effects of blast exposure on the visual system. The test subjects were exposed to a peak static pressure of 138 kPa (20 psi) for 6 milliseconds. It was found that primary blast waves are capable of damaging the optic tract and retina. One potential mechanism is via retinal cell damage and anterograde degeneration of fibre bundles along the visual pathway. Another suggested mechanism by which optic tract damage occurs is by the reflection and translations of waves through the skull or body. When a blast occurs, the eyes may even be displaced from their sockets, leading to degeneration of neurons via shearing forces. In another study, mice were exposed to blast wave pressure of 300 kPa (43.5 psi) each day for 3 successive days in a compressed air-driven shock tube [54]. It was found that after 30 days there was activation of retinal glial cells, loss of neurons, inflammation and an increase of phosphorylated tau in the retina. When assessing for cognitive or motor deficits changes there were no noticeable differences in performance, however these may be seen after exposure to greater pressure over a longer period.

### Mechanism of injury to cortical parts of visual pathway

Vision may also be lost through damage to the other visual pathway elements, for example the optic chiasm, optic tract, optic radiation and the primary visual cortex in the occipital lobes of the brain [55]. Some of the TBI visual symptoms which were previously mentioned, e.g. blurred vision, are very likely due to visual processing issues. Over half of the brain’s circuitry is involved in vision perception, processing and controlling eye movements and so an injury to the brain can impact any of these vision-related aspects (Table 1).

**Table 1:** Summarisation of the types of injury that follow TBI which may lead to visual dysfunctions and vision impairment

Injury	Features of injury
Outer eye/globe	Can be caused by open globe injuries such as perforation, penetration, intraocular foreign bodies and rupture from exposure to blasts Can also be caused by exposure to peak overpressure from blasts directed at the globe
Inner eye/globe	Retinal ganglion cell death can be caused by exposure to high blast pressures, as well as blunt impacts. Retinal ganglion cell death can occur when there is damage to the optic nerve as well. Retinal scarring due to elevated blood pressure and retinal haemorrhaging. This is caused by exposure to high impact force from blast TBI
Optic nerve	Optic nerve damage may be direct or indirect Direct TON is usually caused by penetrating injuries from shrapnel from explosives. Indirect TON is usually caused by blunt impact to the head so there is transfer of distal forces and energy from the forehead to the optic nerve; or exposure to peak overpressure from a blast
Occipital lobe and visual cortex	Impact to ventral and dorsal pathways leading from primary visual cortex as a result of mild TBI. TBI directly to the back of the head, or TBI to the front of the head which results in the brain moving back and forth inside the skull may result in occipital lobe damage

*TBI* traumatic brain injury; *TON* traumatic optic neuropathy

Damage to the primary visual cortex (V1), which is probable after a TBI, can result in visual field defects [56]. V1 receives visual input and is involved in the early stage of vision processing. Alnawmasi et al. [57] studied the effect of mTBI on the dorsal (control of actions) and ventral streams (identification of objects) that are central to cortical vision processing. The hypothesis is that visual information leaves the occipital lobe via these two streams that run parallel to each other to the parietal lobe (dorsal) and temporal lobe (ventral). Mild TBI was found to equally impair processing of both streams, as detected by psychophysical measurements and as a result, form and motion visual processing are impacted [57].

The complex nature of the brain and nervous system as well as complexity of TBI mean that every TBI case is unique. There are a multitude of manners in which a TBI can progress, and it depends on the type of TBI itself, the cause and severity and the individual too. The treatment, management and rehabilitation for a TBI patient will be dependent on the case itself (Table 1).

## Effect of TBI aetiology and severity on vision

The U.S. Blinded Veterans Association (BVA) reported that of those servicemen who were evacuated from field operations due to injuries from IED, 14.9% had penetrating eye injuries and visual dysfunctions associated with TBI [58]. According to the Defense and Veterans Brain Injury Center (DVBIC) data, it is estimated that between 2000 and 2017, the number of TBI occurrences without direct eye injury, but with clinical visual impairment was 76,900. There were 4394 annual incidences of TBI reported where there was visual impairment but no direct injury to the eye [59].

Typical vision-related issues which can arise from a TBI are photophobia, diplopia, blurred vision, vision loss or decline, optic nerve injuries [60, 61]. The results of a meta-analysis on visual symptoms following a TBI found that accommodative dysfunction, convergence insufficiency and visual field loss were common [62].

The visual symptoms which may occur following TBI can be influenced by severity and/or aetiology. One of the first studies that emphasised the need for a focus on eye and vision care following TBI was conducted by Goodrich et al. [63]. This study looked at visual function in patients experiencing polytrauma due to blast injuries in comparison to other causes, e.g. motor vehicle accidents, assault and falls, gunshot and/or shrapnel wounds. All 50 patients in the study were reported to have suffered from TBI. It was found that there was a greater occurrence of vision loss in those that had blast-related injuries (52%) than in those that were injured via other causes (20%). Overall, visual complaints were reported by 74% of the patients, and moderate to total blindness was found in 38% of all cases. The rate of vision loss was 2.5 times greater in those who had a blast injury compared to those who did not. The severity of the blast injuries was not reported but it is probable that they were moderate to severe as the subjects were polytrauma patients. Stelmack et al. [64] reported that in their study of 88 TBI patients from the Hines Polytrauma Network Site, 75% had self-reported with visual symptoms, however TBI severity within this group was not specified.

Goodrich et al. conducted another study to determine the impact of injury aetiology on the frequencies or types of visual dysfunctions [65]. Records from patients who suffered from a blast-related TBI were compared to those from patients who experienced non-blast related TBI. It was found that the aetiology of injury had no real effect on the frequencies or types of visual dysfunctions. Light sensitivity and saccadic dysfunction were the only areas where there was a significant difference between the groups. The blast injury group showed a greater percentage of subjects with light sensitivity compared to the non-blast related group, i.e. 67% vs. 33%. Both groups shared the commonly occurring symptoms found in other studies e.g. deficiencies in oculomotor functions, vergence and accommodation [66].

Dougherty et al. [67] attempted to determine the effects of injury severity. This retrospective study focused on visual dysfunction as a result of blast-related TBI in those who served in Iraq. The key finding was that with an increase in injury severity the likelihood of being diagnosed with a vision related disorder also increases. The authors also reported that within 1 year of a blast injury only 11% of the TBI patients in this study had visual dysfunctions. Blindness and low vision were reported in only 0.47% of the TBI group. However, this study was subject to limitations as it did not use any visual screening tools, clinical evaluation or self-report measures to assess visual problems for the purpose of this study. Instead, the authors relied on existing medical records, and so it is possible that the true number of patients with visual dysfunction is greater.

Unfortunately, visual symptoms may persist for years following a mTBI without being addressed. Magone et al. [68] investigated the prevalence of visual problems in a sample of 31 patients who had been in Iraq or Afghanistan and had suffered from a mTBI for over 12 months. Patients had been diagnosed with blast-induced mTBI but had no direct injuries to the eyes. Overall, 68% of patients had visual complaints with the most common being photophobia and reading difficulties; there were no reports of vision impairment.

A retrospective study involving TBI patients from the Polytrauma Rehabilitation Center (PRC) and Polytrauma Network Site (PNS) programs of the VA Palo Alto Health Care System compared both injury severity and aetiologies [66]. The patients in the PRC (*n* = 68) had moderate to severe TBI and those in the PNS (*n* = 124) had mTBI. In the PRC population, 84% had blast-related injuries and the remaining 16% had non-blast-related TBI, whilst in the PNS group 90% had blast-related injuries. The PRC group had a greater percentage of patients with the following visual complaints: pursuit and saccadic dysfunctions, fixation instability, strabismus, visual field loss, which may suggest that certain visual dysfunctions are more frequent in those with a more severe injury. The PNS group however showed greater percentages for convergence and accommodative insufficiency. When comparing the mode of injury however, there were a number of differences in findings. For some, the frequencies of visual complaints were greater for blast injuries, but for other complaints, numbers were higher for non-blast injuries. Most visual complaints, however, appeared as a result of both blast and non-blast injuries. Both the PRC and PNS groups had patients with legal blindness, although there was a greater percentage in the PRC group (12.7% vs. 1.6%). Legal blindness in the PNS group was only due to blast injuries, but legal blindness was more frequent for those with non-blast injuries in the PRC population. Total blindness was only found in the PRC group; however, this was only found in 2 patients (3.2%) and it was solely due to blast injures. This suggests that it is the more severe TBIs which result in complete sight loss. Limitations of this study include: not taking into account any medication, discrepancies in the numbers between the PRC and PNS cohorts and also between blast and non-blast.

The 2019 meta-analysis found no difference in the prevalence of the four outcomes (accommodative dysfunction, visual acuity, visual field loss and vergence insufficiency) between blast and non-blast injuries [62]. The authors also attempted to stratify results by severity (mild and moderate-to-severe). However, this presented with challenges as not all studies specify severity, or there were numerous participants with varying degrees of severity. In addition, some studies referred to the head injuries as concussions which is often interchangeably used with mTBI, but again the definition of concussion was not specified. Overall, they could only vaguely conclude that some outcomes (accommodative dysfunction and convergence insufficiency) were not affected by severity. In terms of vision loss, there were 3.2% cases in the moderate-to-severe category in comparison to 0.0% in the mild group. Yet, only one study with moderate-to-severe was included in this analysis. Other factors such as diagnostic criteria and study design may also influence the prevalence of visual outcomes.

Along with cause of injury and severity, the stage at which vision problems present themselves should also be taken into account [69]. Many of the aforementioned studies use data from patients receiving polytrauma care from the VA months or years after being medically discharged following the injury, i.e. in the chronic stage of the injury [69]. The number of studies that focus on the early stages of the injury (acute and sub-acute) are very limited. As a consequence, Capó-Aponte et al. [69] investigated the prevalence of visual disturbances during the subacute stage (between 15 to 45 days following injury). The study obtained data from two groups of U.S. military personnel which were matched according to age. The control group consisted of subjects that were deployed but had no history of TBI, whilst the other group consisted of active-duty military personnel that had experienced a mTBI within the last 45 days. Results show that it was mainly the oculomotor functions that were affected. Medication taken soon after the injury for symptoms of depression, pain and PTSD are known to have ophthalmic side effects which can affect the assessment of oculomotor functions that require effort such as convergence. A later study by Capó-Aponte et al. [70] further investigates the visual dysfunctions and symptoms present at different stages (acute/subacute, chronic ≤1 year, chronic > 1 year) after both blast and non-blast related mTBIs. It was found that there is very little difference in symptoms and dysfunctions between the different stages following an injury, and as seen in some previous studies there are few differences between the blast and non-blast groups.

It is important to note that throughout this section, much of the literature cited has not clearly stated whether or not visual dysfunction and/or impairment in the case of blast-TBI occurred via the cortical route, or by exposure to blast fragments, or by pressure from the blast wave itself. It is evident that a TBI can be responsible for many ocular and visual symptoms and dysfunctions, including total sight loss. With the rise in acts of terrorism such as terrorist bombings in civilian settings, studies that have primarily focussed on veterans and military personnel, such as the ones referred to in this review, can be insightful in determining the appropriate treatment and care procedures required following traumatic events.

## Effect of sex and gender

There is a vast amount of literature that looks at TBI in the military and its consequences, however a reoccurring limitation to these studies - much like many other areas within medicine - is that there is significant underrepresentation of women in the study cohorts. In the last hundred years women have been involved in the military in many forms, but it is in the most recent years that they have had frontline combat-based, combat-support and combat service support roles. By 2043, the percentage of women in the U.S. veteran population is expected to rise from 10 to 16% [71]. Despite this, however, due to small sample sizes, there is little data that illustrates the true impact of military related brain injuries on them. Even when looking at those who play close contact sports where TBI is prevalent, most of the data is related to men. TBI research in general almost exclusively includes men [72]. With such a skew towards one gender, the whole picture of military-related injuries is incomplete. As more women are participating in military roles, there is a greater need to understand the differences in outcomes of TBI in women. Although men represent the majority of those with TBI, women are more likely to have worse outcomes [73, 74]. Furthermore, women are statistically more likely to face domestic abuse and violence [75]. Many victims of domestic violence experience blunt force trauma to the head - a well-known cause of TBI. This is an area of growing interest in TBI research.

There are gender-based differences in TBI symptomology and prognosis. In sports for example, women are at greater risk of having a concussion with prolonged symptoms and worse outcomes [76]. Meltzer et al. did a study looking at frequent pain or headache, negative affect, and fatigue in men and women with and without a TBI [72]. It was found that women with a history of mTBI reported with more frequent pain/headaches. The study also suggests that men are more likely to manage their symptoms through externalising behaviours, e.g. substance abuse and impulsivity, and may not report feelings of fatigue or negative mood as a result of feeling uncomfortable in reporting due to expectations of gender norms. This contrasts with women who are more likely to internalise their symptoms.

There is little information regarding how visual outcomes after a TBI may be affected by sex. Secondary analysis of self-reported symptoms showed that 57.9% of women in a retrospective data set of those with long-term TBI outcomes had reported visual problems [77].

TBI studies featuring women are limited, but there are even fewer studies relating to women in the military who have sustained TBI. According to Iverson et al. [78], OEF/OIF women veterans who have deployment-related TBI were 2.0 times more likely to be diagnosed with depression, 1.3 times more likely to be diagnosed with non-PTSD anxiety, and 1.5 times more likely to have PTSD with comorbid depression compared to men. Yaffe et al. [79] found that in their female veteran study population aged 55 and over, having a history of TBI raised the risk of having dementia by 50%. Female service members also reported with more post-concussion symptoms than men following a mTBI [80]. Sexual assault is not uncommon within the military. Military sexual trauma is defined as sexual assault or sexual harassment that takes place during active duty, active or inactive training [81]. Washington et al. found that women who faced military sexual trauma were less likely to utilise care provided by the Veterans Health Administration (VHA), which could mean missing out on vital medical care [82]. This could greatly impact on women being able to receive appropriate care for the physical and mental health needs which may arise, not just from being subject to sexual violence but also if they had any TBI-related issues.

There are discrepancies between what animal and human studies find regarding the effect of sex on TBI outcomes. Some animal studies indicate that female hormones may offer a neuroprotective effect but in human studies females tend to fare worse than males [74]. It is clear that sex is only one of a number of factors (e.g. severity, age, cognitive status) that need to be considered, and these factors may interact and influence outcomes.

As well as members of the military population, prisoners are also susceptible to TBI. Prisoners are also at greater risk of disability following a TBI as a result of previous exposure to substances and mental health difficulties which may have reduced cognitive reserve [83]. When looking at the female prison population in a New Zealand prison, 94.7% (36 out of 38) presented with a history of TBI and 21% of female offenders in a French prison self-reported with TBI [84, 85]. In a study done by The Disabilities Trust, 64% of the offenders in a UK female prison cohort report a history of TBI and 62% attribute their TBI to domestic violence [86]. Again, the TBI history in this population was established using a self-reporting tool (Brain Injury Screening Index).

Most studies which refer to sex and gender often restrict their cohorts to biological sexes and use gender and sex interchangeably without considering the effects of sex and gender separately. The Meltzer study was unique in that it also considered alternative genders [72]. This study included transgender and non-binary individuals, albeit it was a small minority. These individuals reported with higher scores for negative affect and fatigue in comparison to men and women (all without TBI). As the call to allow transgender people to join the military continues to gain support in the U.S., and there are transgender individuals in the UK military, they increasingly become an important community of the population which should also be included in military population studies.

There are many areas which remain unexplored regarding the role that gender plays in the pathways leading to, and the progression, recovery and outcome of military related TBI. The information relating to sex effects on vision outcome following a TBI, whether they are in the military/veteran population or not, is even more limited. Research into TBI in female prison populations and female sports is only just beginning to gain traction. These are populations which we can look to for examples of factors contributing to TBI in women. In the present review, we have only touched on a select few points in an already limited pool of literature. We propose that sex and gender effects on TBI outcome and TBI-related vision outcome in the military population should be explored in a further, more focused review. It should be noted that, in addition to gender effects, the influence of race and ethnic background is another factor worth considering as these can also influence diagnosis and prognosis.

## Treatment of TON

There is currently no approved standardised treatment therapy for TON [87], but observation of injury progression is recommended. As well as observing and monitoring, current treatment options at the moment consist of a course of high-dose steroids, surgical decompression (relieves pressure around optic nerve), and combination of steroids and surgery [47]. The use of high-dose corticosteroids remains controversial and it has been found that it may increase the risk of death following a head injury [88]. There are arguments for not offering any treatment at all as it may be ineffective or worsen the condition, and, as already mentioned, there is greater potential for recovery from this type of optic neuropathy than from direct TON.

## Treatment and management of TBI

With proper management and care, many patients with a single mild TBI can recover within 3 months [89]. Acute management of TBI in the field involves firstly identifying whether there is any intracranial injury that requires urgent surgical treatment, before checking for a possible TBI and directing the injured individual towards appropriate care for their symptomology. The approach to evaluation of TBI in the military setting depends on the country, i.e. the UK and Canada consider the symptoms displayed in order to judge if a TBI may have occurred, whereas the U.S. and the Netherlands require a medical evaluation to take place if an individual has been exposed to a blast, or any other type of event that could potentially cause a TBI [12]. The general consensus in TBI management is that early detection and intervention are key to resolving symptoms and preventing a worse outcome. In the military setting this can be difficult as there may be avoidance or inability in seeking help. To keep track of how a TBI is progressing, neurocognitive testing coupled with clinical examinations is useful. The UK, for example, uses the Rivermead Post Concussion Symptoms Questionnaire, while the U.S. and Canada use the Military Acute Concussion Evaluation (MACE) [12]. The results can be helpful in determining when to return to duty.

There are no TBI-specific treatment or management guidelines for the TBI related visual problems. Management is based on existing treatments and protocols which are applicable to the specific conditions and symptoms being displayed [12]. Photophobia, for example, may be managed by using lenses that filter specific light wavelengths [90]. Visual acuity deficits may be treated with spectacles which have specific tints and prisms [91].

## Eye-tracking as a potential diagnosis method for TBI

Diagnosing TBIs, particularly mTBIs, can be challenging due to a number of reasons for example, the fact that many of their symptoms overlap with PTSD, and the fact that many of the initial acute symptoms, such as loss of consciousness and post traumatic amnesia, are resolved after the patient rests for a sufficient period of time [92]. In addition, clinical attention may be more focussed and directed towards injuries that are more visible. There may also be an inclination towards assuming that conditions such as PTSD are more likely to be present, and so creating a bias towards diagnosing and/or treating other conditions and potentially missing TBI completely. Furthermore, current neuroimaging modalities are not sensitive nor accurate enough to determine whether a mild TBI has occurred [93]. Research in this area is mainly focused on finding a blood biomarker that can detect a TBI and/or advancing neuroimaging techniques that are more sensitive and accurate, however at present there is still no gold standard diagnostic tool or technique for concussions.

Visual dysfunctions, such as abnormal eye movements, are a common result of TBI or concussion. Therefore, the ability to analyse eye movements to detect abnormal ones has more recently been considered as a diagnostic option. This is not a completely novel approach as the King-Devick test has been used for sideline concussion/TBI testing in sports, along with other concussion assessments, it too relies on analysing eye movements [94]. Samadani et al. have developed a new tool to aid in the assessment of mTBI [95, 96]. It relies on tracking and analysing eye movements as the patient is made to watch a 220 s video on a screen placed in front of them. A positive result corresponds to the eye movements that may be present in someone with a concussion or without, and a negative result from the device corresponds to eye movements that are not related to a concussion. The technology relies on the fact that intact vergence is required to focus and maintain binocular vision. Vergence refers to how well the two eyes are bifoveally fixating on the same target during a visual task. Following a TBI or concussion, one of the consequential visual dysfunctions that may occur is insufficient vergence. The device records the centre position of each pupil over time as the patient watches the video in order to track eye-movements of each eye [96]. The device is not intended as an alternative to a CT scan nor should it be used as a stand-alone diagnostic tool [97]. Although the method recently received Food and Drug Administration (FDA) approval in the U.S., it is still not a widely used technique, most likely due to cost and the need for expert analysis of results.

## Conclusion

**Fig. 1:**
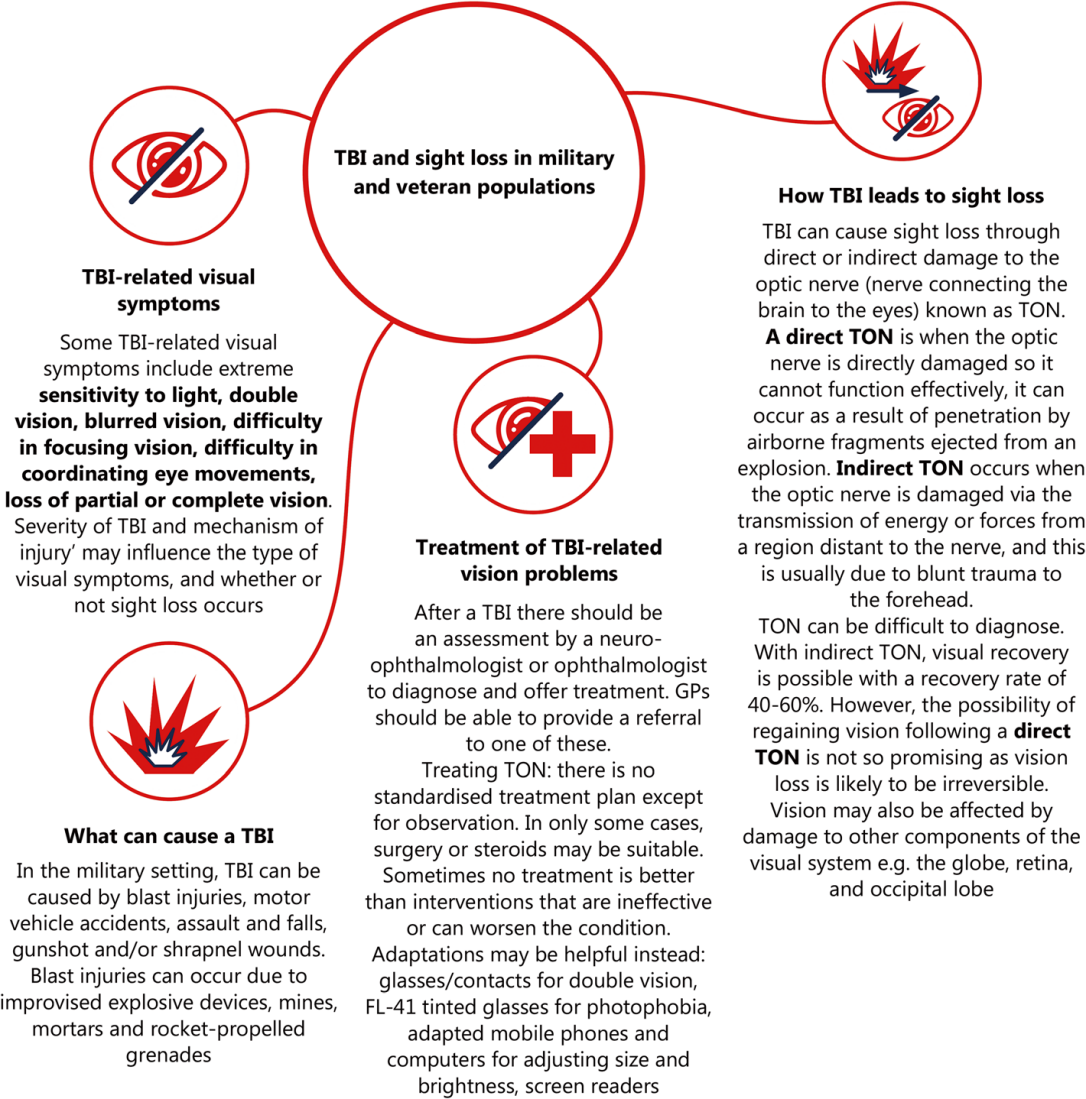
Summary of TBI and sight loss in military and veteran populations. *TBI* traumatic brain injury, *TON* traumatic optic neuropathy, *GPs* general practitioners

TBI are common amongst both the civilian and military population. Throughout history visual dysfunctions and impairments have been a significant problem for military personnel and veterans due to their more regular exposure to blasts in the field. Fig. 1 shows a summary of TBI and sight loss in military and veteran populations. Injury to the visual system may occur through direct damage to the eye, for example through penetration of the globe by fragments from the explosion site, or by primary blast wave pressure that can damage the retina. Visual dysfunction and impairment may also occur via a more cortical path where the blast waves damage the optic nerve and parts of the brain responsible for vision. Blasts are not the only cause of vision problems, as there have been numerous cases where partaking in sports activities, training activities, or having a motor vehicle accident have resulted in loss of functionality of the eyes. Performing primary repair immediately after an ocular injury in the case of penetrating injuries from explosion fragments, for example, could help in preserving vision. It is more difficult to preserve vision when there has been direct damage to the optic nerve. The issue with TBI, specifically mild ones with no obvious wound to the head, is that it can be easily missed. Such injuries may be more likely to go undiagnosed when a soldier is in the field and cannot be assessed immediately by medical staff, for example, after an explosion has just occurred. Identifying if a TBI has occurred may be instrumental in early action intervention to prevent loss of vision. Tracking eye movements, along with current diagnostic methods, may contribute to an early indication of potential vision problems before they may get irreversibly worse.

Eye injuries and head injuries are not a new phenomenon in war and conflict, although they are becoming more commonplace in the civilian setting with the rise in acts of terrorism and other blunt force trauma injuries. Regardless of which setting we are considering, improving our understanding of vision preservation, and mitigating the effects of TBI is imperative. In the military and veteran population, there are also other factors that may compound the effects of vision loss and combat-related vision loss such as PTSD, dementia, hearing loss, and serious illnesses and disabilities. Ultimately, the loss of vision greatly impacts, not only general physical wellbeing, but also the quality of life and psychological wellbeing of any individual.

Limitations of our selection and inclusion criteria are that much of the data available is retrospective, which means relying on existing medical records where there may be inaccuracies in recording and missing data, e.g. if a patient has had multiple TBI but these have gone unrecorded, and TBI severity is commonly missed out. Medical classifications, screening and diagnostic tools may differ from country to country, meaning that TBI diagnoses, and definitions may also differ. Another issue, which may be more specific to the military population, is that TBI symptoms may be misconstrued for PTSD symptoms due to the general association between military and PTSD, and the overlap of PTSD and TBI symptoms. Moreover, an individual may have both PTSD and TBI, and symptoms of both can be present and difficult to disentangle. This may also particularly occur in cases where there is no obvious head wound to signify a TBI. Furthermore, a common issue with TBI studies that assess long term outcomes is low follow-up rates due to patient attrition [98].

## Data Availability

Not applicable.

## Abbreviations

BVA: Blinded Veterans Association
cFPI: Central fluid percussion injury
CSF: Cerebrospinal fluid
CT: Computed tomography
DVBIC: Defense and Veterans Brain Injury Center
FDA: Food and Drug Administration
IED: Improvised explosive devices
IOP: Intraocular pressure
MRI: Magnetic resonance imaging
mTBI: mild TBI
OCT: Optical coherence tomography
OEF: Operation Enduring Freedom
OIF: Operation Iraqi Freedom
PERG: Pattern Electroretinography
PNS: Polytrauma Network Site
PRC: Polytrauma Rehabilitation Center
PTSD: Post traumatic stress disorder
RGC: Retinal ganglion cells
RNFL: Retinal nerve fibre layer
TBI: Traumatic brain injury
TON: Traumatic optic neuropathy
UK: United Kingdom
U.S.: United States
